# Electrical current modulation in wood electrochemical transistor

**DOI:** 10.1073/pnas.2218380120

**Published:** 2023-04-24

**Authors:** Van Chinh Tran, Gabriella G. Mastantuoni, Marzieh Zabihipour, Lengwan Li, Lars Berglund, Magnus Berggren, Qi Zhou, Isak Engquist

**Affiliations:** ^a^Laboratory of Organic Electronics, Department of Science and Technology, Linköping University, Norrköping 60174, Sweden; ^b^Wallenberg Wood Science Center, Department of Science and Technology, Linköping University, Norrköping SE-601 74, Sweden; ^c^Division of Glycoscience, Department of Chemistry, Royal Institute of Technology, AlbaNova University Centre, Stockholm 106 91, Sweden; ^d^Wallenberg Wood Science Center, Department of Fiber and Polymer Technology, Royal Institute of Technology, Stockholm 100 44, Sweden

**Keywords:** wood, transistor, electrochemistry, PEDOT:PSS, conductivity

## Abstract

The nature of mass transport in plants has recently inspired the development of low-cost and sustainable wood-based electronics. Herein, we report a wood electrochemical transistor (WECT) where all three electrodes are fully made of conductive wood (CW). The CW is prepared using a two-step strategy of wood delignification followed by wood amalgamation with a mixed electron-ion conducting polymer, poly(3,4-ethylenedioxythiophene)–polystyrene sulfonate (PEDOT:PSS). The modified wood has an electrical conductivity of up to 69 Sm^−1^ induced by the formation of PEDOT:PSS microstructures inside the wood 3D scaffold. CW is then used to fabricate the WECT, which is capable of modulating an electrical current in a porous and thick transistor channel (1 mm) with an on/off ratio of 50. The device shows a good response to gate voltage modulation and exhibits dynamic switching properties similar to those of an organic electrochemical transistor. This wood-based device and the proposed working principle demonstrate the possibility to incorporate active electronic functionality into the wood, suggesting different types of bio-based electronic devices.

## Significance

The orthotropic 3D microstructure has recently promoted wood as a template for applications in wood-based energy and electronic devices. Different varieties of electroconductive wood are widely reported; however, modulating the wood’s electrical conductivity without changing its chemical composition has not been done and remains challenging. In this work, we present an approach to preparing conductive wood (CW), in which the electrical conductivity can be modulated using an external potential. This has resulted in a transistor where all three terminals are made of conductive wood and which can be operated continuously at the selected conductivity without being limited by, e.g., saturation effects. We expect this device and concept will be a stepping stone for the development of wood-based electrical components.

As we step into the era of green technologies, there will be an increasing distinction between complex, nanoscale electronics on one hand and simple, large-size or large-area electronics on the other hand, the latter with special functionalities like biosensing, biointegration, biodegradability, etc. Bio-based materials will be the underpinning for the development of these functionalities. During the last decades, cellulose, lignin, conducting polymers, and other organic or bio-based materials have emerged as potential templates or active components in various electrical devices (1, 2). Among these materials, wood stands out when it comes to materials that have the potential for ion transport and regulation (3). Several studies have shown that the unique three-dimensional (3D) microstructures of wood lumina are ideally designed for mass transport in the electrodes of electrochemical devices (1–4). As a result, wood has been carbonized or functionalized with conductive materials for applications in supercapacitors, batteries, and electrochromic screens (2–6). After removing lignin, the open pathways generated along the wood fibers have been shown to provide promising ion transport channels in nanofluidic devices (7). Besides that, wood was also explored in other electrical systems such as triboelectric nanogenerators and electrical magnetic shielding (8, 9). These achievements indicate that wood has a huge potential for energy and electronic technology. Wood is orthotropic, and the directionality can provide advantages for organic transistor performance. However, among many reported devices using a wood template, there is, to the best of our knowledge, yet no report of an electrical transistor made of wood or even of electronically induced modulation of conductivity in wood-based conductors.

In order to transform wood to be an active component in a conventional transistor [semiconductor transistor or organic electrochemical transistor (OECT)], tunable electrical conduction is required. The conductivity can be induced by either wood carbonization or wood modification with conducting polymers such as polyaniline, polypyrrole, or poly(3,4-ethylenedioxythiophene)–polystyrenesulfonate (PEDOT:PSS) (5, 10, 11). However, modulation of the conductivity of carbon conductors is not possible by electronic or electrochemical measures, which rules out carbonized wood as a transistor channel material. This leaves wood modified with conducting polymers, which will be the material system of choice in the present study. Earlier attempts for “wood-based transistors” include studies focused on utilizing wood and its derivatives as a nonconductive substrate(11) for templating a conventional transistor. In this direction, cellulose paper is a common choice as it has good flexibility and in some cases also high transparency (11, 12) Cellulose fibers also show potential as a structural component in the electrolyte of an electrolyte gate transistor (12). Although cellulose has been used for silicon-containing transistors (13), transistors not based on silicon technology could in the future reduce electronic waste and be biodegradable. In a recent attempt, Li et al. reported a wood-based membrane that could regulate ion transport through modulation of an external voltage (7). For this purpose, the authors have coated a gate electrode layer of silver metal on the wood surface and applied the working principle of a gating transistor for regulating the ion movement in the membrane. This results in a kind of ionic transistor, where the authors have proved that ionic current can be regulated at the nanoscale of the wood scaffold. Such a device has interesting potential but will also be limited by the need to transfer between electrical current and ionic current at the electrodes. The anticipated buildup of ionic charge or electrochemical reaction products at the interfaces will inhibit the possibility to operate the device in a steady state for longer periods of time. For the prolonged operation of a transistor, it is required to rely upon modulation of electrical conduction (7). Therefore, we need an approach that might utilize the wood ionic conductivity but also includes sufficient and tunable electrical conduction in wood for the actual transistor channel. Building on recent progress in creating PEDOT:PSS-based conductive wood (CW) (10), the principle of operation for OECTs should be a suitable candidate that could provide such tunability. The OECT builds upon a transistor channel that is electrically conductive and that can be electrochemically modulated using ionic transport between the transistor channel and a gate electrode (14, 15). Since both these transport mechanisms can be realized within a wood template, a fully wood-based OECT should be possible to construct. The device is not expected to have high performance compared to conventional transistors as, in this work, the primary focus is to prove the hypothesis that electrical conductivity of the CW can be modulated by using an external potential. The result of this attempt is a wood transistor, in which all electrodes are made of CW. We believe this device concept will be a good example for encouraging the use of earth-abundant and sustainable resources in specific electrical applications.

**Fig. 1.**
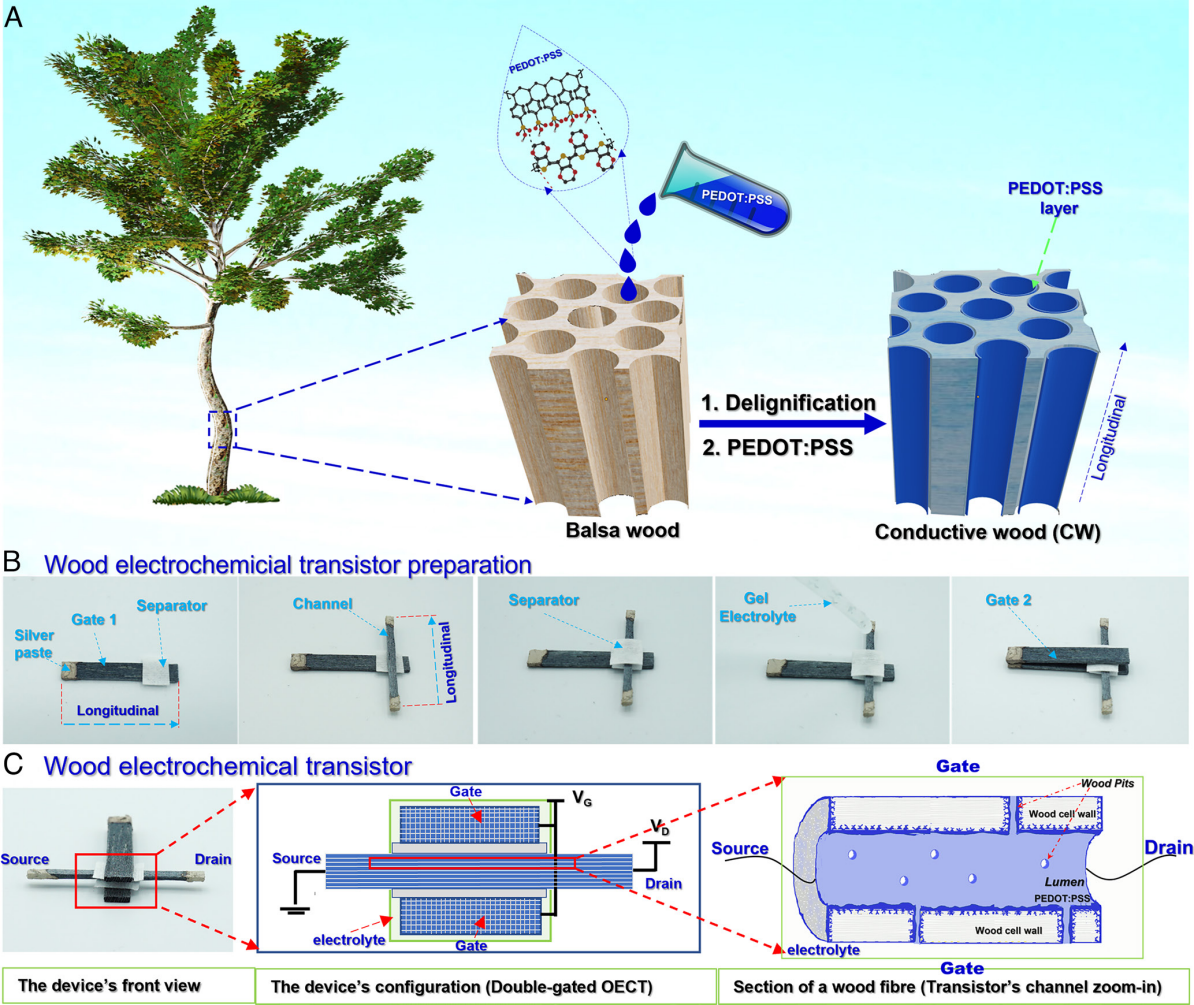
The schematic diagrams of (*A*) conductive wood preparation and (*B*) the wood electrochemical transistor fabrication processes. (*C*) From *Left* to *Right*: Front view photograph of a WECT, front view of the WECT configuration, and section of a wood fiber (conceptual view of transistor’s channel zoom-in) showing PEDOT:PSS-coated cell wall and electrolyte-transporting lumen.

As shown in Fig. 1, the wood electrochemical transistor (WECT) was made from three pieces of CW. The CW was prepared using a two-step strategy of wood delignification and wood amalgamation with PEDOT:PSS (Fig. 1*A* and *SI Appendix*, Fig. S1). Hardwood balsa was selected owing to its high strength, low density, and high permeability, as well as its relatively homogeneous structure with less difference between earlywood and latewood regions compared to softwood (10, 16). In a preliminary test, we observed that balsa performed better than birch or ash in preparing high–CW electrodes (*SI Appendix*, Fig. S2). The conducting polymer was selected due to its excellent tunable electrical conductivity shown in numerous examples to provide a successful OECT channel (14, 17, 18) for, e.g., neuromorphic signaling and chemical sensing applications (14). The preparation method enables the formation of a beneficial microstructure of PEDOT:PSS in the wood scaffold (Fig. 1*A*). This microstructure not only provides electrical conductivity but also leaves room to utilize the wood 3D architecture for ionic transport (17). The CW, after that, was used to construct a WECT with an approach that is adopted from the fabrication of a double-gate OECT.

The assembly of a WECT is explained in Fig. 1*B*, where two pieces of CW (longitudinal × tangential × radial = 30 mm × 5 mm × 1 mm) were used as the bottom and the top gates (denoted as WECT-Gate). Another piece of CW (longitudinal × tangential × radial = 30 mm × 2 mm× 1 mm) was used as the transistor channel (WECT-Channel), while a cellulose/polyester lab tissue and a gel–electrolyte mixture were used as separator and electrolyte, respectively. The final WECT transistor is configured as a double-gate OECT, in which the main operation process (reduction/oxidation of the conductive polymer) happens at the microscopic scale in the lumina that form the transistor channel, Fig. 1*C*.

## 1. Results and Discussion

To be employed as an active component in a transistor, the wood must have sufficient electrical conductivity. In some studies of wood coated with conducting polymers, it has been found that wood pretreatment methods including wood delignification can enhance the conductivity (6, 10, 19, 20). In this work, the balsa wood was delignified before being impregnated with the PEDOT:PSS suspension.

**Fig. 2.**
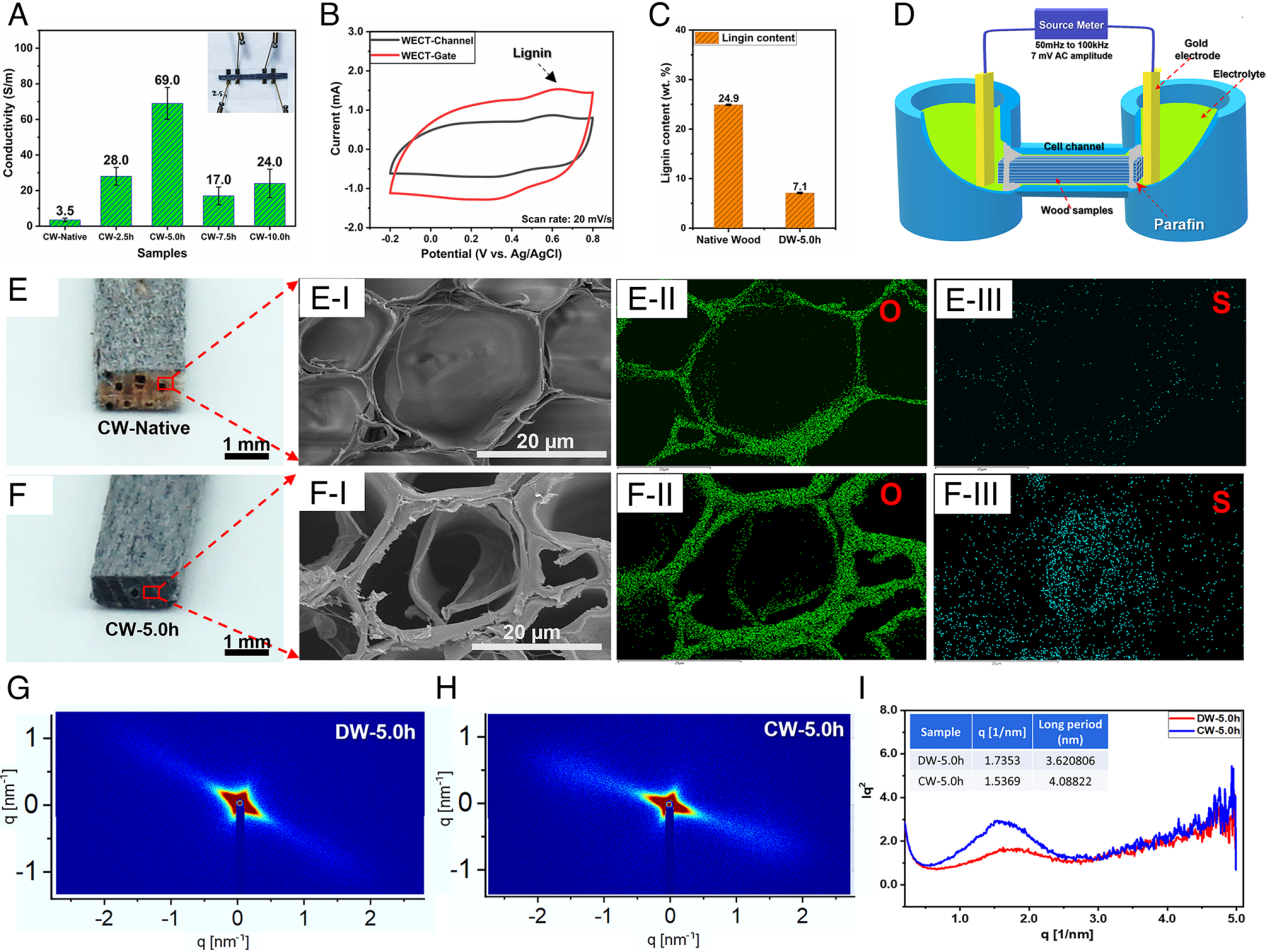
(*A*) The conductivity of CW samples fabricated using different delignification times (*Inset*: four-point probe measurement setup). (*B*) Cyclic voltammetry of the WECT-Channel and WECT-Gate samples. (*C*) Lignin content of Native and DW-5.0h. (*D*) Illustration of the ionic conductivity measurement setup. (*E* and *F*) are the cross-sectional images of CW-Native and CW-5.0h samples, respectively. (*E*, *I*) and (*F*, *I*) in turn are the cross-sectional SEM images taken at the middle of supercritically point-dried CW-Native and CW-5.0h samples. (*E*, *II*) and (E, *III*) are the EDX elemental mapping of oxygen and sulfur in the corresponding SEM images of CW-Native. (*F*, *II*) and (*F*, *III*) are the EDX elemental mapping of the oxygen and sulfur in the corresponding SEM image of CW-5.0h. (*G* and *H*) are the SAXS patterns of DW-5.0h and CW-5.0h, respectively. (*I*) The analyzing results of 1D SAXS spectra of DW-5.0h and CW-5.0h (*Inset*: the calculated figures of DW-5.0h and CW-5.0h).

The effect of wood delignification on the CW conductivity was examined by varying the wood delignification time from 0.0 (native wood) to 10.0 h, with results shown in Fig. 2*A*. We see that the CW-5.0h has the highest conductivity (69.0 ± 9.0 Sm^−1^), while the CW-Native shows the lowest value of 3.5 ± 1.0 Sm^−1^. In native balsa wood, liquid transport occurs mainly in the lumina of vessels. With the removal of lignin, wood is expected to gain a higher porosity in the cell walls, and the pits in the cell walls are open (21), which makes the lumina of fibers and parenchyma cells available to transport PEDOT:PSS suspension (22). In addition, diffusion pathways are also opened up in the middle lamella and cell wall corners in the delignified wood (DW) (23). As a result, the fiber lumina, which are the dominating structures in balsa wood, can be coated with PEDOT:PSS. The improved PEDOT:PSS diffusion results in a higher electrical conductivity in the wood. However, when the delignification time is longer than 5 h, we discovered that the wood fiber cells collapse as the softened cell walls fall onto each other, causing a “compacting of the cellular structure.” This is evident in a reduced sample thickness in the DW-7.5 h and DW-10.0 h samples (*SI Appendix*, Table S1). The collapsing hinders efficient polymer diffusion in the structure (24), thus leading to a drop in the conductivities from 69 ± 9.0 Sm^−1^ of CW-5.0h to 17.0 ± 5.0 Sm^−1^ of CW-7.5 h. There is a slight increase to 24.0 ± 8.0 Sm^−1^ for CW-10.0 h, but we judge this to be due to natural sample variations, and we focus on the large difference compared to the CW-5.0h sample. Based on these results and differently from other reported studies (24, 25), we found that there is in fact an optimum delignification time. This is due to an optimum lignin content resulting in an optimum level of sample integrity. Accordingly, we selected 5.0h as the optimal delignification duration, and the corresponding CW (CW-5.0h) was used for the fabrication of the WECT. The WECT-Channel and WECT-Gate were made of CW-5.0h in sizes of 30 mm × 2 mm × 1 mm (longitudinal × tangential × radial) and 30 mm × 5 mm × 1 mm (longitudinal × tangential × radial), respectively. Although having a bigger sheet area, the WECT-Gate has a lower conductivity (30.0 ± 4.0 Sm^−1^) than the WECT-Channel (69.0 ± 9.0 Sm^−1^). This probably relates to the poorer access of PEDOT:PSS to the interior parts of the larger wood piece, which has a 2.5 times larger cross-section. One plausible reason for this is that the initial PEDOT:PSS adsorption in the outer parts of the sample may partially block PEDOT:PSS diffusion into the inner parts of the sample. There could also be a delignification gradient contributing in the same way, but we believe this to be less plausible since the good permeability of the balsa wood should ensure homogeneous delignification on the 1–radial mm scale. However, we note there is a potential for improvement in future works, where the effects of the samples’ geometry, the delignification gradient, and the polymer infiltration gradient should be studied (26).

In addition to the electrical conductivity, the electrochemical properties including charge storage capacitance and ionic conductivity are important for understanding the applicability of CW as active electrodes (Gate and Channel) in an OECT. While the device operates, the 3D structure of CW is expected to facilitate a sufficient charge accumulation, which in turn will play a key role in switching the current passing through the CW-based device channel (14).

As shown in Fig. 2*B*, both the WECT-Gate and the WECT-Channel show good capacitive behavior with their CV curves assuming a slightly deviated rectangular shape. The deviation is most probably caused by the redox activity of small amounts of native lignin, remaining in the DW (DW-5.0h), and therefore also present in the CW-5.0h sample (10). The lignin content was determined using the TAPPI T222 om-02 method and amounts to 7.1 ± 0.1 wt% which is significantly lower than the 24.9 ± 0.1 wt% in the native wood (Fig. 2*C*). Both the WECT-Gate and the WECT-Channel show good capacitances of 55.0 ± 5.0 mF and 31.0 ± 4.0 mF, respectively, at the scan rate of 20 mV/s (see the specific capacitances in the *SI Appendix*). The higher capacitance of WECT-Gate is a consequence of its larger size (2.5 times the volume of WECT-Channel). For the operation of OECTs, it is advantageous if the gate electrode has a larger capacitance than the transistor channel(26–28). As an additional observation, we note that for other electrochemical devices including supercapacitors, the capacitance results suggest the CW-5.0h as a potentially useful material (10). Along with the electrodes’ capacitances, the ionic conductivity of the WECT-Channel was also studied to understand its capability for ion-mediated electrochemical conductivity regulation when an external voltage is applied. By using the measurement setup shown in Fig. 2*D*, the recorded ionic resistance of the WECT-Channel (or CW-5.0h sample) is lower than that of DW-5.0h. This implies that PEDOT:PSS has played an important role in lowering the ionic resistance, which in turn means an increase in the ionic conductivity within the CW scaffold. More detailed results (*SI Appendix*, Fig. S3) and further discussion are presented in the *SI Appendix*.

Structural and morphological characterization was performed to map and understand the wood’s morphology and PEDOT:PSS distribution and is presented in Fig. 2 *E* and *F*. By comparing CW-5.0h with CW-Native, we observed a clear distinction in their appearance and microstructure. As seen in Fig. 2 *E* and *F*, where cross-sections from the middle of each sample are shown, CW-5.0h appears dark blue throughout its thickness, while the cross-section of CW-Native reveals its native light brown color. This indicates that PEDOT:PSS has penetrated the entire DW structure but was not able to access the inner parts of the native (lignified) wood. Accordingly, at a microscopic scale as investigated by scanning electron microscopy (SEM), there is no trace of PEDOT:PSS in the cross-section of CW-Native (Fig. 2 *E*, *I* and *III*). In contrast, a PEDOT:PSS layer was seen in the fibers’ lumens (Fig. 2 *F*, *I* and *SI Appendix*, Fig. S4) and vessels’ lumens (*SI Appendix*, Fig. S5) of the CW-5.0h. This observation was further confirmed by Energy-dispersive X-ray analysis (EDX) elemental mapping images, in which sulfur was mostly observed in the wood lumen (Fig. 2 *F*, *III*). It should be noted that the PEDOT:PSS thin film in Fig. 2*F* was visualized by applying supercritical point drying instead of air-drying (*SI Appendix*, Fig. S4) as the liquid CO_2_ causes detachment of the polymer from the wood cell wall, making it clearly visible. In the pristine samples used in WECTs, the PEDOT:PSS film only coats the inner surface of the lumens (10), leaving the central section open for electrolyte transport. A similar coating phenomenon is expected to happen at the ray cells, contributing to transverse electrical transport and helping to create 3D electrical interconnection in the wood structure. With the observed microstructure thus formed, conceptually illustrated in Fig. 1*A*, PEDOT:PSS is expected to promote both the electron and ion transport through the 3D structure of CW-5.0h and in the WECT-Channel.

In addition to the structure observed in SEM images, the small-angle X-ray scattering (SAXS) measurements shown in Fig. 2 present another insight into the distribution of PEDOT:PSS in the wood cell wall of CW-5.0h. The two-dimensional SAXS patterns of DW-5.0h and CW-5.0h are presented in Fig. 2 *G* and *H*). Anisotropic streaks were observed, but they only show small differences between the two samples. We further analyzed the one-dimensional (1D) data (Fig. 2*I*) to determine the correlation length, which means the center-to-center distance of the cellulose fibrils. Our calculation suggests that CW-5.0h has a slightly larger correlation length (29) than that of DW-5.0h (≈4.0 ± 0.10 nm compared to ≈3.55 ± 0.15 nm) which could be an indication of penetration of PEDOT:PSS polymer chains in between the CW-5.0h wood fibers. A control measurement indicates that DMSO only has a minor contribution to this effect (cf. *SI Appendix*, Fig. S6). Although we are unable to quantify the amount of PEDOT:PSS inside the wood cell wall, this result suggests that some of the polymer is localized there; however, the amount should be small compared to the amount covering the cell wall. The presence also has some effects on the wood fibers’ arrangement, which was studied by wide-angle X-ray scattering (WAXS) and presented in *SI Appendix*, Fig. S7. The CW-5.0h sample has a smaller Herman’s orientation factor of the 200 crystal plane compared to DW-5.0h, which means the alignment of cellulose fibrils is disturbed, probably as a result of the partial impregnation with PEDOT:PSS. To further investigate the interaction between wood fibers and PEDOT:PSS, ATR-FTIR measurements were carried out for both CW-5.0h and DW-5.0h (*SI Appendix*, Fig. S8). The discussion on FTIR results (*SI Appendix*) suggests that PEDOT:PSS has interactions with the wood fibers, which facilitated the amalgamation of these components in the composite of CW-5.0h (30–32).

Finally, mechanical properties (tensile strength and Young’s modulus) were measured for native, delignified (DW-5.0h), and CW (CW-5.0h) and reveal that the CW is similar in tensile strength and stiffness to the original balsa wood (cf. *SI Appendix*, Fig. S9). Taking all the collected evidence of electrical/electrochemical and structural properties into account, CW-5.0h was selected as a good candidate for forming electrodes for the wood transistor.

### 1.1. WECT.

OECTs can be constructed with a single gate electrode or with double gates on either side of the transistor channel, as shown schematically in *SI Appendix*, Fig. S10 *A* and *B*, respectively. A double-gate configuration is beneficial when the transistor channel dimensions are large since it provides better and faster access for ion transport to all parts of the transistor channel. Here, for a WECT device with a 1-mm-thick channel, the double-gate structure would thus be advantageous. This is experimentally proven by comparing the switching performance of both configurations (cf. *SI Appendix*, Fig. S10 *C* and *D*). In view of this result, double-gate transistors were selected as the standard configuration for further investigations.

**Fig. 3.**
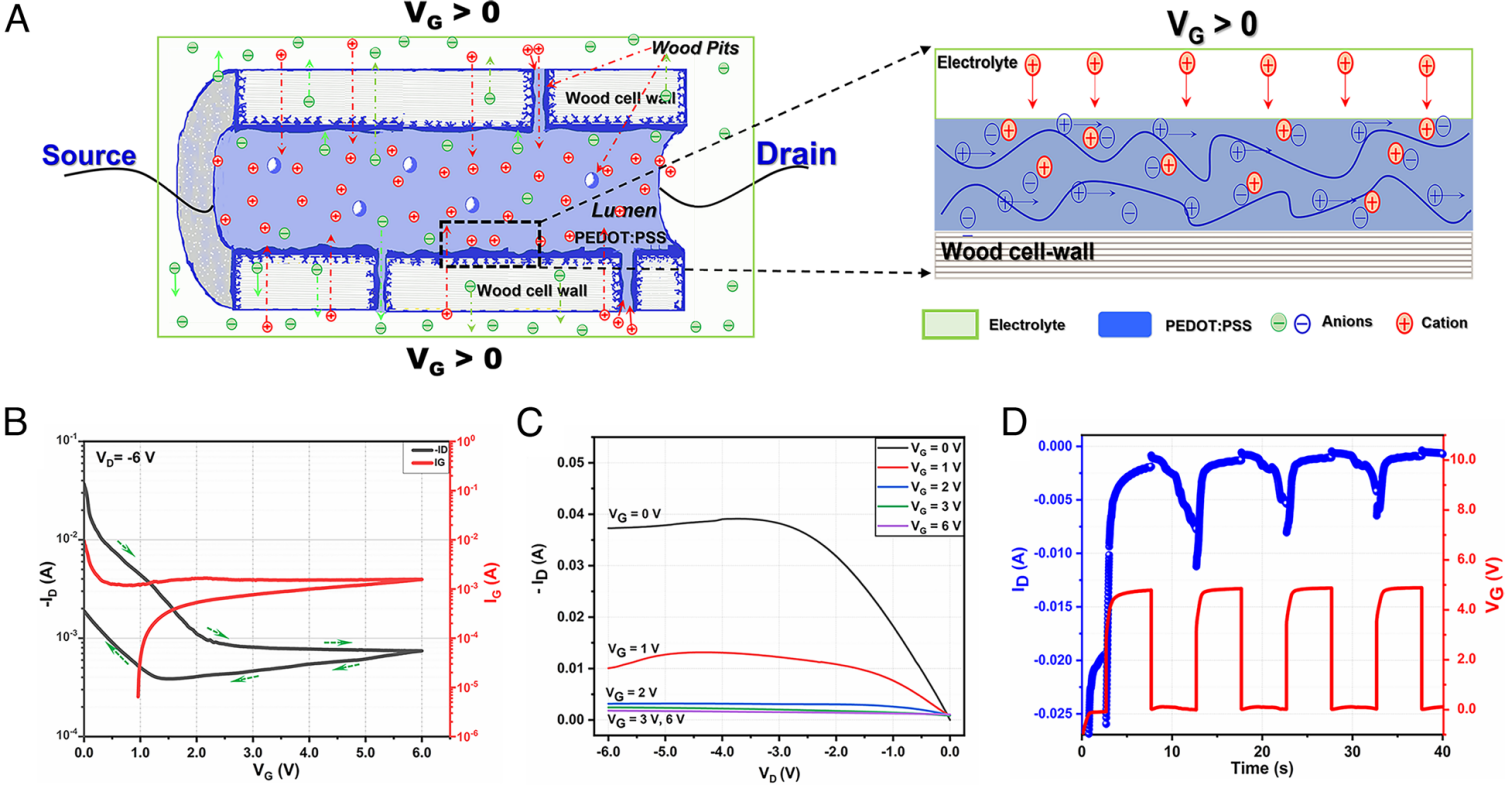
Wood electrochemical transistor (WECT). (*A*) The speculated operation mechanism with a focus on part of a single wood fiber with the cell wall and lumen, (*B*) the transfer curve, (*C*) the output curves at different gate voltages, and (*D*) the dynamic switching characteristics at the frequency of 100 mHz. (Note: Each measurement was carried out on different devices.)

As illustrated in Fig. 1*B*, a double-gate WECT is structured with the two gate electrodes positioned on the top and bottom sides of the transistor channel. Both the WECT-Gate electrodes and the WECT-Channel are made from 1-mm-thick CW-5.0h. Although 1 mm is much thicker than the ordinary thickness of a conventional PEDOT:PSS-based OECT [less than 1 µm (26, 27, 33)], still the device operates like an ordinary p-type OECT. The current passing through the WECT-Channel is defined as the drain–source current (I_D_). At zero gate voltage (V_G_), the transistor channel is open, and the transistor is ON, whereas by applying a gate voltage of 6.0 V, the channel becomes fully reduced, and the transistor is in the OFF state. Fig. 3*B* shows the transfer curves of the device in which the ON/OFF [I_D_(V_G_ = 0)/I_D_(V_G_ = 6.0 V)] current modulation reaches 1.7 orders of magnitude (50 times) for the forward sweep. In comparison with the ON/OFF ratio (hundreds to 10^5^) of conventional PEDOT:PSS-based OECTs (14, 26, 33), the ratio of 50 is small but reasonable for a transistor with a combination of high electrode thickness and limited conductivity. In the same figure, it is observed that the transistor is switched off when V_G_ reaches ≈2.5 V. The switching process is repeatable, which is shown in three consecutive switching runs presented in *SI Appendix*, Fig. S11.

A tentative operation mechanism at a microscopic level of the WECT is illustrated in Fig. 3*A* which is based on the switching performance and the WECT-Channel’s morphology observed in Figs. 1*C* and 2. A section of one wood fiber was selected to describe the working mechanism as it could represent the current modulation principle in the entire WECT-Channel (10). Before applying any potential to the gate electrodes (V_G_ = 0 V), the WECT is in its ON state. Upon applying V_G_ > 0, the WECT is gradually switched to the OFF state due to the electrolyte cations being driven out from the electrolyte toward the wood cell wall surface where PEDOT:PSS is mainly localized and held at negative potential. Here, an electrochemical reaction takes place where the cations compensate the counter anions (PSS^−^), and the PEDOT^+^ is reduced to its nonconductive form PEDOT^0^ (15). As a result, the conductivity of the WECT-Channel is decreased.

Output measurements are carried out to provide additional information about the performance of WECTs. Here, the drain–source voltage (V_D_) was swept from 0 to −6.0 V, while V_G_ was increased in steps from 0 to 6.0 V. In Fig. 3*C*, the obtained I_D_–V_D_ curves are shown. At V_G_ = 0 V and V_G_ = 1 V, we see that I_D_ increases linearly with increasing V_D_ and reaches a plateau at around V_D_ = −3.5 V. After that, I_D_ undergoes a slight decrease when the V_D_ level is increased up to −6.0 V. The decrease is probably related to either a slight reduction of PEDOT:PSS at such a high voltage range or a small current leakage between the drain and the gate. In the output curves where V_G_ is stepped to values higher than −2.0 V, the transistor is in the OFF mode, and such behavior is not observed. This is fully in agreement with the characteristics of ordinary OECTs.

Although the WECT is expected to be slow, we also examined the dynamic switching capability of the device. In dynamic switching measurements, a function generator was sourcing V_G_ as a square wave switching between 0 and 5.0 V at 100 mHz, while a constant V_D_ of −6.0 V was applied. From the graph shown in Fig. 3*D*, we see that repeated dynamic switching is fully possible, although not fast. The main part of the OFF switching happens in around 1 s, which has to be considered as good under the circumstances (a 1-mm-thick transistor channel). The ON switching is slower and is not fully completed in 5 s, which is probably the reason why we see a decreasing ON/OFF ratio as the measurement in Fig. 3*D* progresses. For full ON/OFF dynamics, a lower frequency than 100 mHz would have to be used. With these dynamic switching properties, the WECT is not suitable for conventional electronic circuits but is probably an interesting candidate for wood-integrated applications ranging from electrochromic displays to simple logic circuits responding to sensor input.

## 2. Conclusions

A transistor made of CW was successfully demonstrated. This result proves that it is possible to modulate the electrical conductivity of the electroactive wood by applying an external voltage. The WECT operates according to the same principle as a double-gate OECT, where the two gates and the transistor channel are made of delignified wood, made conductive (69 Sm^−1^) by the formation of a PEDOT:PSS layer in the lumina of the wood structure, in particular the fiber lumina. The current modulation occurs through electrochemical oxidation/reduction of PEDOT, with a measured ON/OFF ratio of up to 50 times. Although the device performance is poorer than the common PEDOT:PSS-based OECT, the WECT proves the principle and shows that there is a possibility to transform wood into a functional transistor by utilizing its oriented and hierarchical 3D structure, thereby introducing the possibility to control and regulate the electronic current in CW. We also believe there are possibilities for improvement by either optimizing the conductivity of wood or manipulating the device configuration. Since the mechanical stability of the transistor electrodes is as good as the original balsa wood, strong and self-supporting devices could be readily constructed. In view of the large interest in exploratory research concerning bioelectronics, bio-based electronics, and plant electronics, this device and its working principle might be a stepping stone toward different applications in those fields.

## 3. Experimental Section

### 3.1. Materials.

Balsa (Ochroma pyramidale) veneers with an oven-dried density of ~0.22 g cm^−3^ were purchased from Material AB (Sweden). Sodium chlorite (NaClO_2_, 80%), sodium chloride (NaCl, 99%), and dimethyl sulfoxide (DMSO, 99%) were received from Sigma Aldrich. PEDOT:PSS (Clevios PH1000, water suspension with ≈1% solid content) was purchased from Heraeus, Germany. Carbon fibers, paraffin wax, silver paste, and carbon paste were purchased from Sigma Aldrich and used as received. Blue gel (250 g) was purchased from Cefar-Complex, Sweden.

### 3.2. Wood Delignification.

Balsa wood veneers were cut in the size of 30 mm × 10 mm × 1 mm (longitudinal × tangential × radial). The veneers were delignified at 80 °C in a NaClO_2_ (1.0 wt%) solution in acetate buffer for different reaction times: 2.5 h, 5.0 h, 7.5 h, or 10.0 h. The obtained DW samples were correspondingly named DW-2.5h, DW-5.0h, DW-7.5h, and DW-10.0h.

### 3.3. CW Preparation.

The DW samples were dried under an ambient atmosphere and cut into smaller pieces. Samples with dimensions of 30 mm × 2 mm × 1 mm (longitudinal × tangential × radial) were used to prepare the CW-based transistor channel, while samples having a dimension of 30 mm × 5 mm × 1 mm (longitudinal × tangential × radial) were used to prepare the CW–based gate of the WECT. These DW pieces were thereafter impregnated in a PEDOT:PSS suspension (100 g of PEDOT:PSS suspension mixed with 6 g of DMSO) before being oven-dried at 75 °C to achieve the CW (see *SI Appendix*, Fig. S1 for a visual diagram). The final CW products were obtained after mechanically removing all the aggregated polymer layers on the surface of the dried samples. Corresponding with the DWs, the obtained CWs were named CW-2.5h, CW-5.0h, CW-7.5h, and CW-10.0h, respectively. The PEDOT:PSS-coated native wood (CW-Native) was prepared in a similar approach, in which native wood was cut in a specific size before being impregnated in the same PEDOT:PSS suspension to achieve the CW. All CWs used as the WECT-Channel and the WECT-Gate were dried and stored in a controlled environment before being used for the device fabrication and measurement.

### 3.4. WECT Fabrication.

The fabrication of a double-gate WECT is presented in Fig. 1*B*, where the two gate electrodes are set perpendicularly to the channel electrode. One gate is on the top surface and the other one is placed under the bottom surface of the transistor channel. The channel and gates are separated by a cellulose-based tissue paper before dropping the electrolyte mixture on the crossing area of the electrodes (Fig. 1*B*). The electrolyte is prepared by mixing 2 mL blue gel (Blågel, Cefar-Complex) with 1 mL NaCl (1 M) and kept for 2 weeks before use.

## 4. Characterization

### 4.1. Morphology and Chemical Composition.

SEM/EDX: The morphology of the wood samples was analyzed by field emission SEM (Hitachi S-4800, Japan) at a low acceleration voltage of 1 kV. The samples were microtomed, dried either in ambient conditions or under supercritical CO_2_, and coated with a platinum/palladium conductive layer using a sputter coater (Cressington 208HR, UK). EDX was performed at an acceleration voltage of 6 kV with an Oxford Instruments, X-MAX N 80, UK.

Lignin and Monosaccharides Content: Klason lignin content was determined by acid hydrolysis according to the TAPPI T222 om-02 method. The samples were analyzed in duplicates. Quantification of the neutral sugars was performed on Dionex ICS-3000 high-performance ion-exchange chromatography (Thermo Fisher Scientific Inc.) after acid hydrolysis. The samples were analyzed in duplicates, and anhydrous factors were used for the monosaccharides (0.88 for xylose and arabinose and 0.90 for glucose, mannose, and galactose). Meier’s correlations were used to calculate the weight percentage of cellulose and hemicellulose.

Leaching experiments were carried out for the WECT-Gate and WECT-Channel by soaking the CW samples in deionized water for 4 d. The results indicate no significant leaching and are presented in the Supporting Information (*SI Appendix*, Fig. S12).

### 4.2. Conductivity Measurement.

The CW was glued on top of 4 chromium/gold electrodes by carbon paste, and the resistance was measured using a 4-probe technique. A Keithley 2400 source meter was used to supply the current to the two outer electrodes and to measure the voltage between the two inner electrodes.

Based on the obtained resistance, the electrical conductivity (σ) of CW samples is calculated using the following equation (10)^:^

[1] $$\sigma =\frac{1}{\rho }=\frac{L}{\mathrm{RA}},$$

where R is the obtained resistance, L is the distance between the two inner electrodes, and A is the cross-sectional area of the specimen.

The sheet resistance of CW samples was calculated using the following equation:

[2] $$R_{s}=\frac{1}{\sigma t},$$

where R_s_ is the sheet resistance, and t is the thickness of the sample.

### 4.3. Electrochemical Measurement.

The CW electrode for electrochemical measurement was prepared following the same procedure as in our previous work. The CW is first connected to carbon fibers using carbon paste before wrapping a part of the carbon fiber with paraffin wax and Kapton tape, respectively. The electrochemical measurement was performed in a three-electrode system configuration using a potentiostat/galvanostat (by BioLogic, SP-200) coupled to a computer. The capacitance of samples was calculated using the formula (34): C=$\frac{1}{v\Delta V}\int_{V1}^{V2}idV=\frac{A}{2\times k\times \Delta V}$ , where i is the charge/discharge current (A), A is the integral area of the CV curve, k is the scan rate (mV/s), and ΔV is the working potential of the discharge process.

### 4.4. Wood OECT Characterization.

Transfer (drain current vs. gate voltage), output (drain current vs. drain voltage), and dynamic switching (drain current vs. time) measurements were conducted using a semiconductor parameter analyzer (*HP/Agilent 4155B*) and a function generator (*Agilent 33120 A*).

### 4.5. SAXS and WAXS Measurements.

SAXS and WAXS measurements were performed on a point-collimated Anton Paar’s SAXS point 2.0 system equipped with a Cu Kα radiation source (wavelength 1.5418 Å and beam size of ~500 µm) and an Eiger R 1M detector with 75 × 75 µm pixel size (at RISE, Sweden). The sample-to-detector distance was set to 576 mm and 111 mm for SAXS and WAXS, respectively. The exposure time of each measurement is 10 min, and they were performed at room temperature with a beam path pressure of about 1 to 2 mbar. The data reduction was performed by using SAXS analysis software (Anton Paar, Graz, Austria).

Please refer to the additional SAXS measurement, which was carried out at the CERMAV-CNRS (France), in the *SI Appendix*.

## Supplementary Material

Appendix 01 (PDF)

## Data Availability

All study data are included in the article and/or *SI Appendix*. Source data and the data used to generate the figures, are available from figshare with the identifier https://doi.org/10.6084/m9.figshare.22325092.v1.
