# Supplementary material for: Electrical current modulation in wood electrochemical transistor
Source: Proc Natl Acad Sci U S A. 2023 Apr 24;120(18):e2218380120. doi: 10.1073/pnas.2218380120 (PMC10160952; doi:10.1073/pnas.2218380120)
Supplement: Supplementary file 1 — Appendix 01 (PDF) [file pnas.2218380120.sapp.pdf]

Supporting Information for

Electrical Current Modulation in Wood Electrochemical Transistor

Van Chinh Tran[1,2], Gabriella G. Mastantuoni[3,4], Marzieh Zabihipour [1], Lengwan Li[4], Lars A. Berglund[4], Magnus Berggren[1,2], Qi Zhou[3,4], Isak Engquist[*1,2]

[1] *Laboratory of Organic Electronics, Department of Science and Technology, Linköping University, 60174 Norrköping, Sweden.*

[2] *Wallenberg Wood Science Center, ITN, Linköping University, SE-601 74 Norrköping, Sweden*

[3] *Division of Glycoscience, Department of Chemistry, KTH Royal Institute of Technology, AlbaNova University Centre, 106 91 Stockholm, Sweden.*

[4] *Wallenberg Wood Science Center, Department of Fiber and Polymer Technology, KTH Royal Institute of Technology, 100 44 Stockholm, Sweden*

[*] *Corresponding author: Prof. Isak Engquist (isak.engquist@liu.se)*

Experiments and Results

The conductive wood's preparation process is demonstrated in the synthetic diagram below:

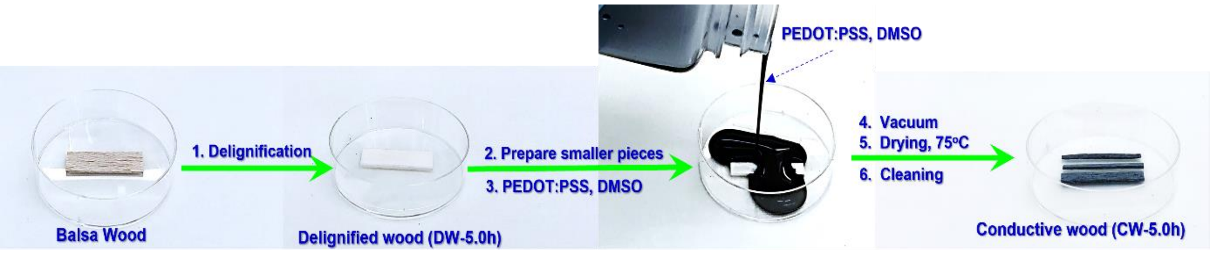

Figure S1: The synthetic diagram of conductive woods.

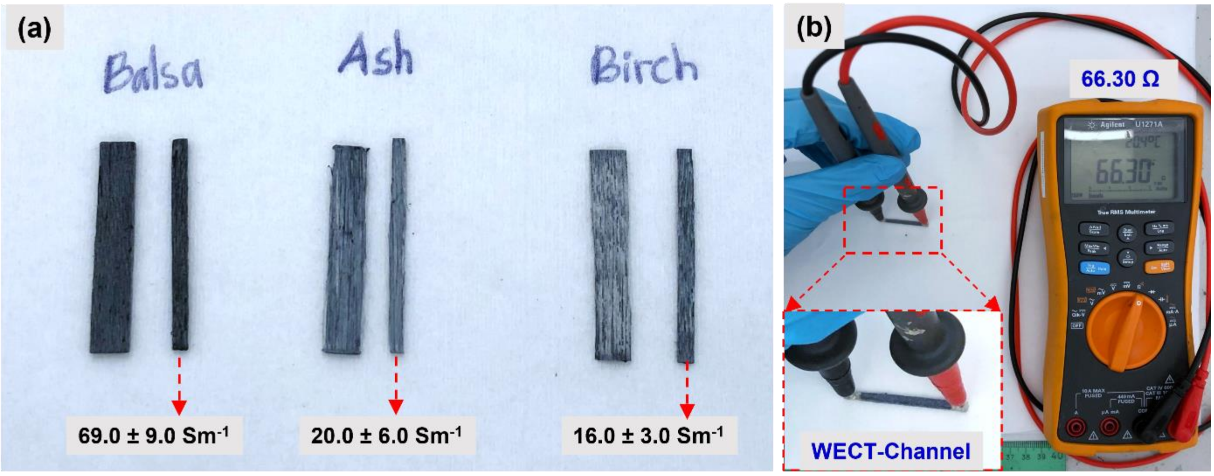

Figure S2: a) The highest conductivities obtained from different types of conductive woods (balsa, ash, and birch) using the same preparation method as in Fig. S1, b) The illustration of WECT-Channel's resistance using a digital multimeter.

Table S1: Samples' thickness

	**Native wood**	**DW-2.5h**	**DW-5.0h**	**DW-7.5h**	**DW-10.0h**
Thickness (mm)	1.14 ± 0.03	1.13 ± 0.03	1.12 ± 0.03	1.07 ± 0.04	1.04 ± 0.03

Electrochemical properties of CW-5.0h (WECT-Gate and WECT-Channel)

The specific volumetric (or mass) capacitances of WECT-Gate and WECT-Channel are 353 ± 32 $mFcm^{-3}$ (or 1.22 ± 0.11 Fg^{-1}) and 461 ± 60 $mFcm^{-3}$ (or 1.78 ± 0.23 Fg^{-1}), respectively, at the scan rate of 20 mVs^{-1}. The volume or mass used for calculation is the volume or the total mass of the electrode.

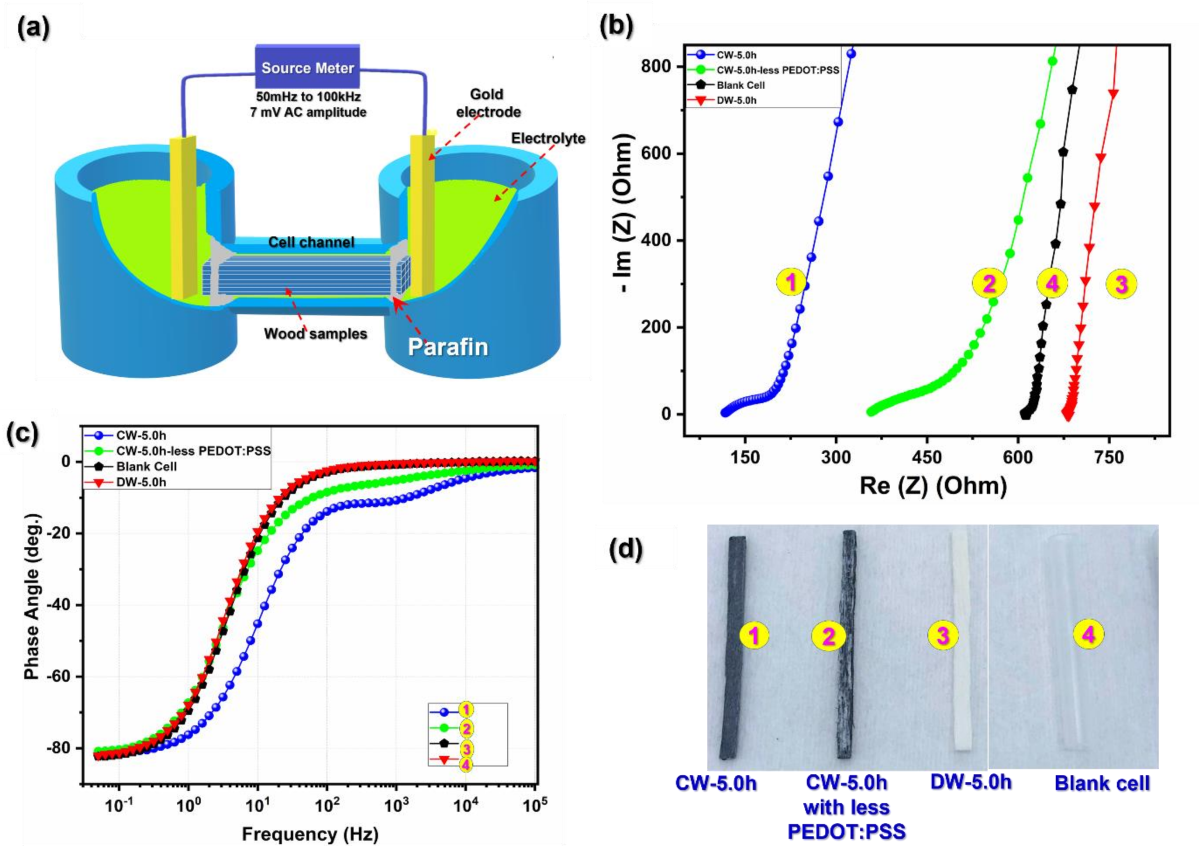

Figure S3: a) Ionic conductivity measurement setup. b) Nyquist plots and c) Bode plots of DW-5.0h, CW-5.0h with different fractions of PEDOT:PSS; and the blank cell channel; and the cell channel-containing PEDOT:PSS; d) All samples used for ionic conductivity measurement. Note: All wood samples were prepared from the DW-5.0h sample, which has an areal size of 2×30 mm^2 (tangential × longitudinal).

- Sample (1) is the CW-5.0h (WECT-channel), which has the PEDOT:PSS content of ≈22.0 wt% (≈5.5 mg/cm^2),
- Sample (2) is the CW-5.0h with a lower content of PEDOT:PSS (≈ 9.0 wt%, or ≈1.9 mg/cm^2).

  The sample was obtained by carefully drop-casting 100µl of the PEDOT:PSS: DMSO suspension onto DW-5.0h (tangential × longitudinal = 2 mm×30 mm) before drying at 75°C in ambient air.
- Sample (3) is the delignified wood (DW-5.0h).
- Sample (4) is the Blank cell. A Blank cell means there is no sample in the cell channel.

The measurement was designed to understand the capability of the WECT-Channel for ion-mediated electrochemical conductivity regulation when an external voltage is applied. For this

purpose, we developed a lab-made two-probe measurement setup (Fig. S3a)(1-3), in which two gold electrodes were used to collect the ionic current passing through a 3 cm-long sample. There was no Ohmic contact between the electrodes and the sample. The electrolyte for measurement is NaCl, 1M, which is one of two components in the gel electrolyte of WECT. The Nyquist and Bode plots of all samples are respectively presented in Fig. S3b and S3c, in which the conductive wood sample (CW-5.0h or WECT-channel) shows a significantly lower resistance than that of the non-electrically conductive sample (DW-5.0h). Indeed, by intercepting the exploitation straight line of Nyquist plots at the low frequencies with the x-axis(1, 3, 4), the resistance of DW-5.0h and CW-5.0h were determined to be 685 Ω, and 200 Ω, respectively. Besides that, the resistance of CW-5.0h is also smaller than the ionic resistance of the Blank-cell, 627 Ω, which did not contain any sample. The interestingly low ionic resistance of CW-5.0h could be attributed to the excellent ionic conductivity of PEDOT:PSS, and probably the polarization of the conducting polymer under the effect of an external voltage[...(5, 6). For further investigation, we have lowered the PEDOT:PSS content in the CW-5.0h and found that the lower polymer content has higher ionic resistance (in Fig. S3b, the resistance of sample (2) is 520 Ω while the resistance of sample (1) is only 200 Ω). These results bring us to the conclusion that the presence of PEDOT:PSS in the conductive wood (CW-5.0h) gives it a higher ionic conductivity than that of the corresponding delignified wood (DW-5.0h).

Structural Morphological properties of CW-5.0h

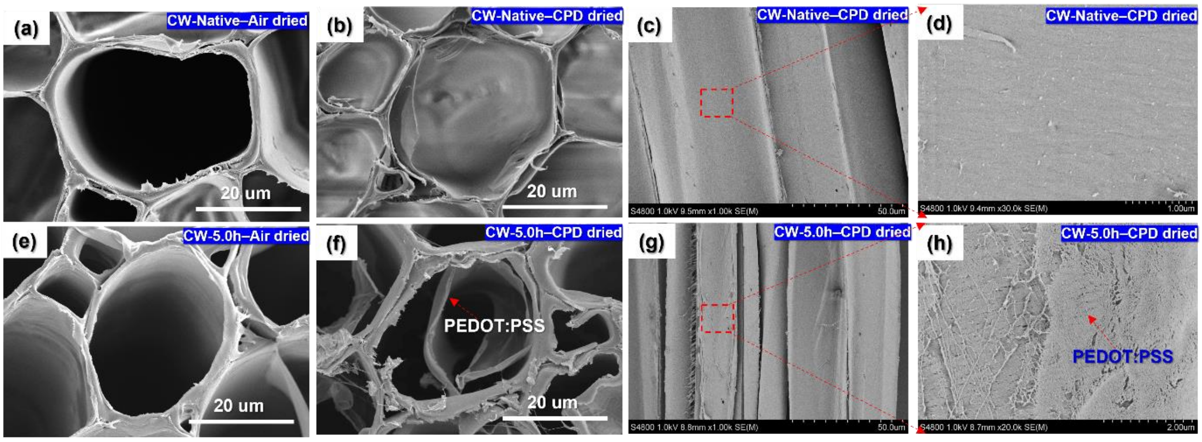

Figure S4: Cross-sectional SEM images of CW-Native in (a) air-dried and (b) CPD-dried samples; (c) and (d) are longitudinal SEM images of CPD-dried CW-Native sample; cross-sectional SEM images of CW-5.0h in (e) air-dried and (f) CPD-dried samples; (g) and (h are longitudinal SEM images of CPD-dried CW-5.0h in the sample.

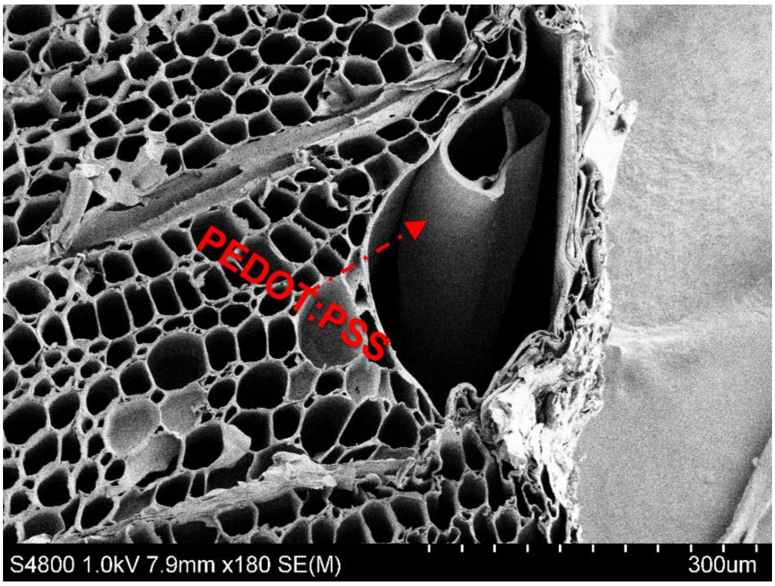

Figure S5: SEM images of the cross-section of CW-5.0h showing the presence of PEDOT:PSS in the lumen of a vessel. The PEDOT:PSS layer can be peeled off during the air-drying process.

Additional SAXS measurement at CERMAV-CNRS (France).

The experimental description: the X-ray machine has a Ni-filtered Cu KR radiation with a wavelength of 1.542 Å equipped with a Philips PW3830 generator operating at 30 kV and 20 mA. The X-ray beam was perpendicular to the wood chip surface. The exposure time was set to 1 h for each sample. The scattering patterns were recorded on Fujifilm imaging plates and read by a Fujifilm BAS-1800II bioimaging analyzer. The 2D X-ray pattern was decomposed into the 1D anisotropic profile and isotropic profile fitted to the pseudo_Voigt function as described previously (7). The correlation length was obtained from the peak position of the Kratky curve of the anisotropic profile.

The measurement results:

As DMSO contributed 5 wt% of the polymer suspension mixture, we prepared a control sample (DW-5.0h/DMSO) by impregnating DW-5.0h in a 5 wt% DMSO solution in water before drying at 75°C in the atmospheric condition. In Fig. S6, the SAXS measurements were carried out for DW-5.0h, the control sample (DW-5.0h/DMSO), and CW-5.0h, of which the obtained correlation lengths are 3.55 ± 0.15 nm, 3.65 ± 0.10 nm, and 4.00 ± 0.10 nm, respectively. The results indicate that both the polymer and DMSO contributed to the increased distance, but the polymer has a major contribution.

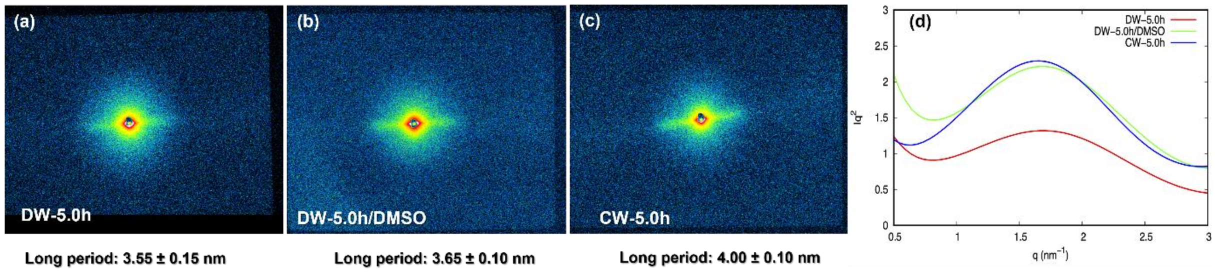

Figure S6: The SAXS patterns of a) DW-5.0h, b) DW-5.0h/DMSO, and c) CW-5.0h, d) the analyzing results of 1D SAXS spectra of DW-5.0h, DW-5.0h/DMSO, and CW-5.0h. Note: the SAXS patterns and 1D spectra were obtained from measurements carried out at CERMAV-CNRS (France), while the reported long period values are the average of results obtained from different samples measured at RISE (Sweden) or at CERMAV-CNRS (France).

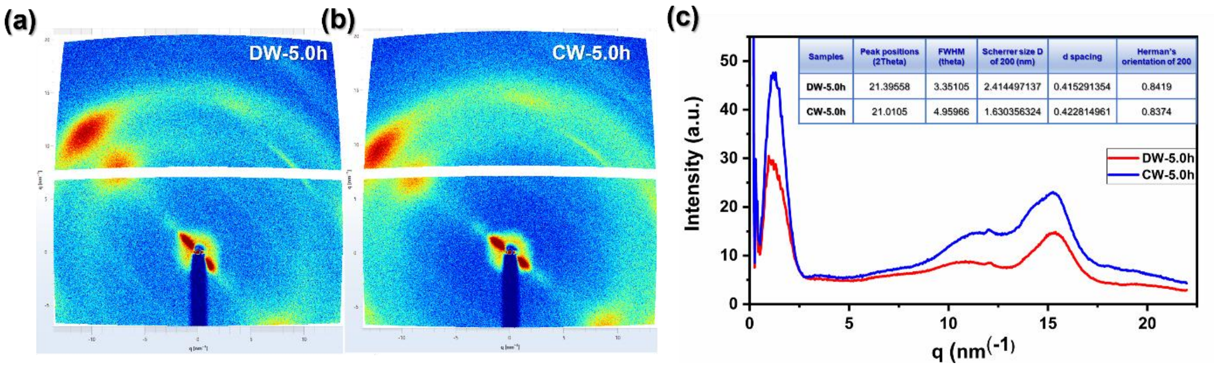

Samples	Peak positions (2Theta)	FWHM (theta)	Scherrer size D of 200 (nm)	d spacing	Herman's orientation of 200
DW-5.0h	21.39558	3.35105	2.414497137	0.415291354	0.8419
CW-5.0h	21.0105	4.95966	1.630356324	0.422814961	0.8374

Figure S7: Wide Angle Xray Scattering (WAXS): a) and b) WAXS patterns of DW-5.0h and CW-5.0h, respectively; c) 1D-WAXS spectra of DW-5.0 and CW-5.0h (inset table: the calculated figures from 1D-WAXS measurement). Note: the results were obtained from measurements carried out at RISE (Sweden).

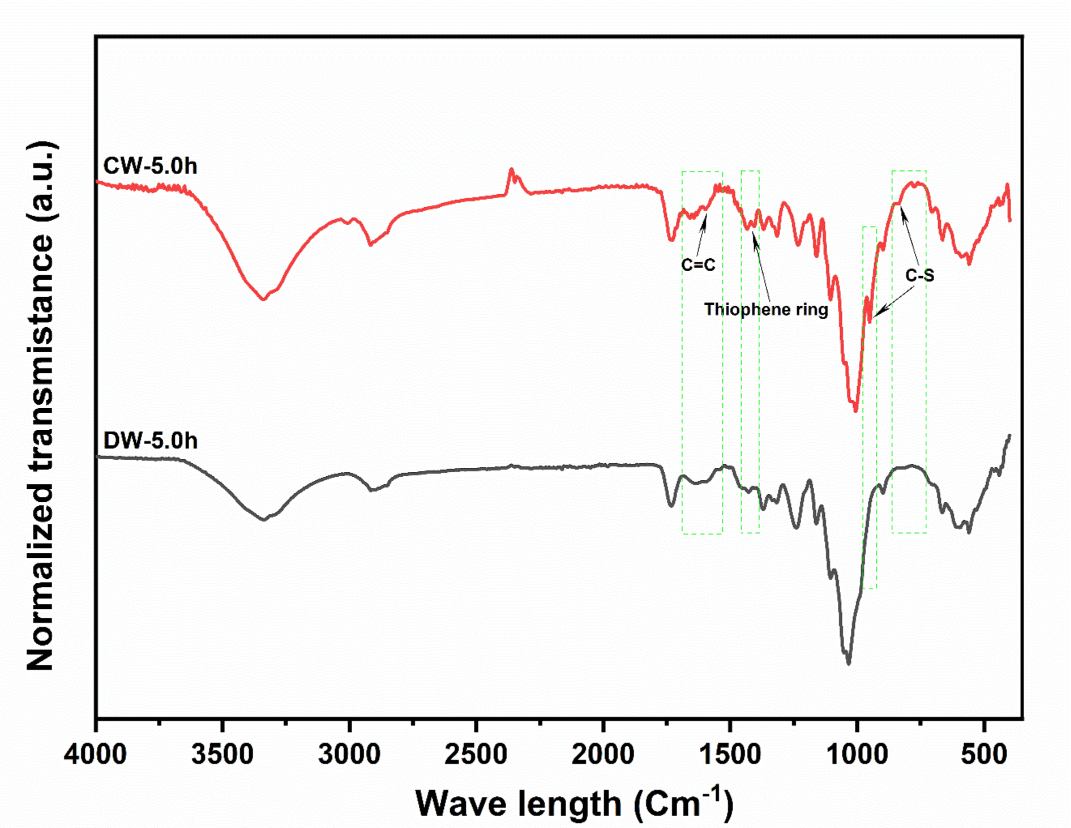

Figure S8: ATR-FTIR spectra of DW-5.0h and CW-5.0h.

In Fig. S8, two samples' spectra show a good correlation as the major peaks of DW-5.0h were repeated at the same position in the spectrum of CW-5.0h. The overlap suggests a good combination between the wood components and PEDOT: PSS. In the spectrum of CW-50h, the existence of the polymer can be recognized by its typical peaks located at 1404 cm^{-1}, 1433 cm^{-1} (C_β-C_β stretching of the thiophene rings)(8, 9), and at 951 cm^{-1}, 901 cm^{-1}, and 833 cm^{-1} (C-S bond in the thiophene ring of PEDOT). An interesting distinction was also seen in the position of the hydroxyl group (3300 cm^{-1}), where the peak in CW-5.0h is broader and has a higher intensity than that of the DW-5.0h spectrum. The broadening peaks can be attributed to the hydrogen-bonding interaction between wood fibers and PEDOT:PSS (10)

Mechanical properties of CW-5.0h.

In Fig. S9, the tensile strength of the samples in the longitudinal direction was not modified during processing and remained stable at around 30 MPa for Native wood, DW-5.0h, and CW-5.0h. Young's Modulus decreased after delignification, resulting in slightly less stiff material as lignin is removed. However, the addition of the polymer brought back the modulus to values similar to the ones of native wood.

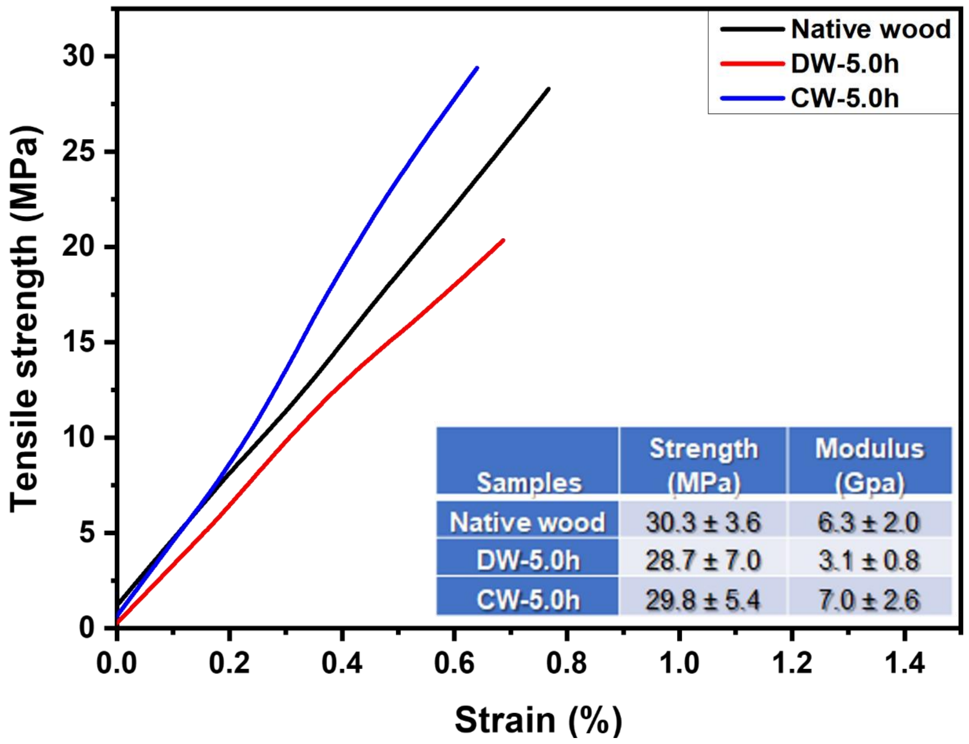

Figure S9: Mechanical properties of wood samples: Typical Tensile strength-stress of Native wood, DW-5.0h, and CW-5.0h (inset: the strength and Young's modulus of the samples).

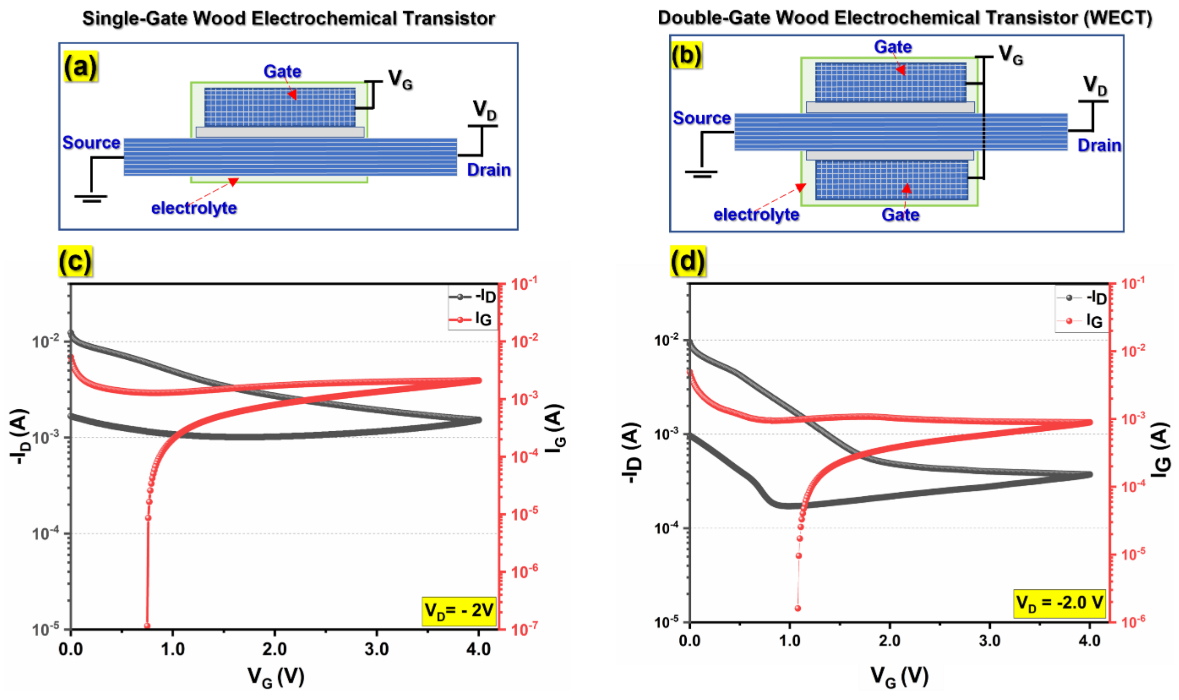

Figure S10: The performance comparison between the single-gate and double-gate configurations: a) and c) the single-gate device configuration and its transfer sweep; b) and d) the double-gate device configuration and its transfer sweep.

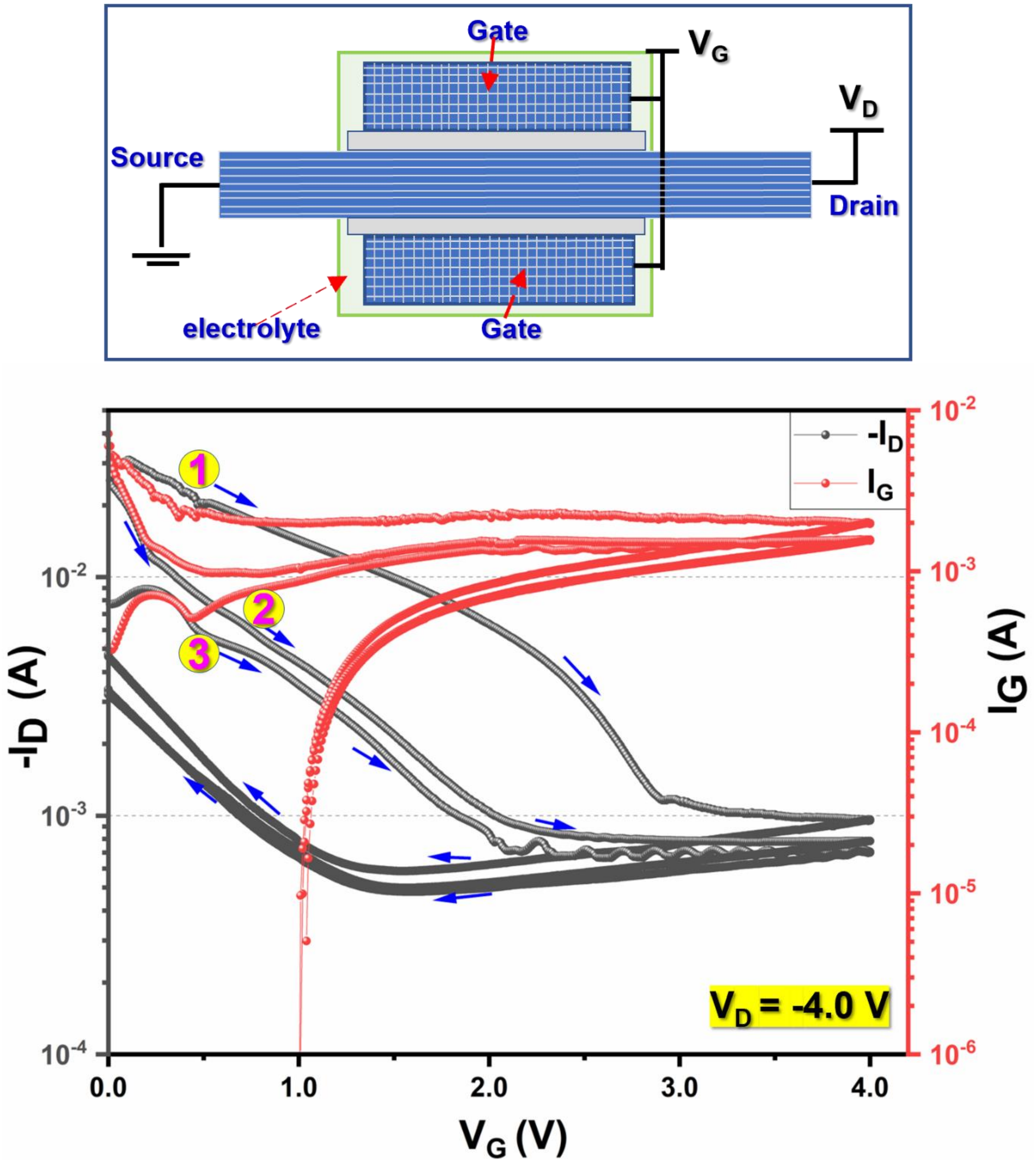

Figure S11: WECT's transfer weeps: (1) the first transfer sweep; (2) the second weep conducted right after the first transfer sweep; (3) the third sweep collected after resting the device for 1 minute.

Leaching experiments:

WECT-Gate and WECT-Channel were soaked in deionized water before recording their appearance and measured mass every day of 4 days test. As seen in Fig. S12, the appearance of both the samples and the solution remains the same over time. The samples' masses were measured and suggested no significant change after soaking in water for 4 days. There is a slight mass reduction after 1 day (≈5wt%), after which the mass remained nearly constant from day 2 onward. The small reduction after day 1 is probably related to the discharge of DMSO from the wood to the water solution. The results suggest that the device electrodes have good resistance to humidity and wet conditions.

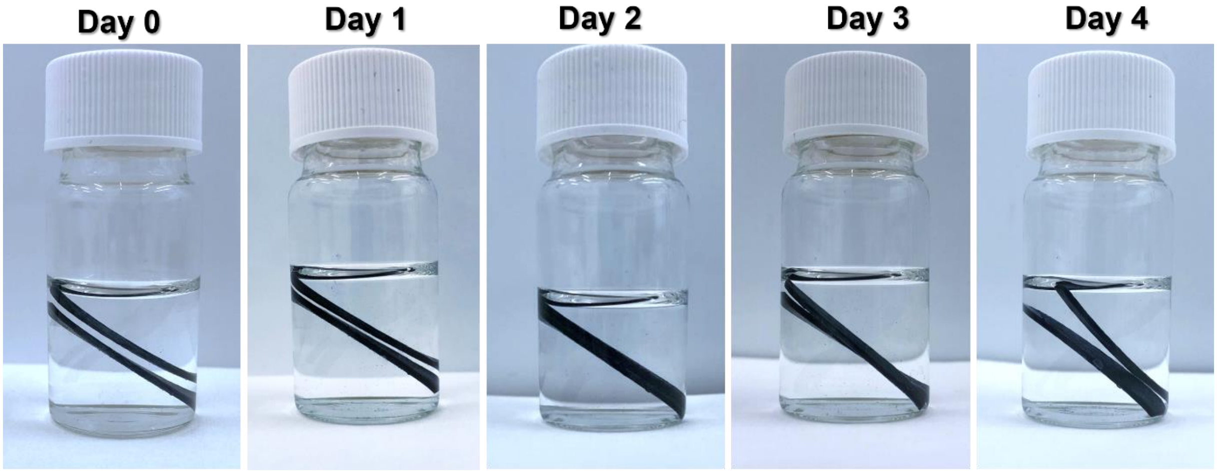

Figure S12. The leaching experiment of WECT-Gate and WECT-Channel electrodes.

References:

1. Y. H. Ye, Y. F. Zhang, Y. Chen, X. S. Han, F. Jiang, Cellulose Nanofibrils Enhanced, Strong, Stretchable, Freezing-Tolerant Ionic Conductive Organohydrogel for Multi-Functional Sensors. *Adv Funct Mater* **30** (2020).
2. W. Kong *et al.*, Wood Ionic Cable. *Small* **17**, e2008200 (2021).
3. R. Inada, T. Okada, A. Bando, T. Tojo, Y. Sakurai, Properties of garnet-type Li6La3ZrTaO12 solid electrolyte films fabricated by aerosol deposition method. *Prog Nat Sci-Mater* **27**, 350-355 (2017).
4. V. C. Tran, S. Sahoo, J. J. Shim, Room-temperature synthesis of NiS hollow spheres on nickel foam for high-performance supercapacitor electrodes. *Mater Lett* **210**, 105-108 (2018).
5. B. D. Paulsen, K. Tybrandt, E. Stavrinidou, J. Rivnay, Organic mixed ionic-electronic conductors. *Nat Mater* **19**, 13-26 (2020).
6. E. Said, N. D. Robinson, D. Nilsson, P. O. Svensson, M. Berggren, Visualizing the electric field in electrolytes using electrochromism from a conjugated polymer. *Electrochem Solid St* **8**, H12-H16 (2005).
7. S. V. Pingali *et al.*, Morphological changes in the cellulose and lignin components of biomass occur at different stages during steam pretreatment. *Cellulose* **21**, 873-878 (2014).
8. S. Funda *et al.*, Correlation between the fine structure of spin-coated PEDOT:PSS and the photovoltaic performance of organic/crystalline-silicon heterojunction solar cells. *J Appl Phys* **120** (2016).
9. Y. Z. Liu *et al.*, Enhanced Dispersion of TiO2 Nanoparticles in a TiO2/PEDOT:PSS Hybrid Nanocomposite via Plasma-Liquid Interactions. *Sci Rep-Uk* **5** (2015).

10. M. Lay *et al.*, Smart nanopaper based on cellulose nanofibers with hybrid PEDOT:PSS/polypyrrole for energy storage devices. *Carbohydr Polym* **165**, 86-95 (2017).
